# Different hypoxic response of human *CAVII* and *Caenorhabditis elegans* homologous genes *CAH-3, CAH-4, CAH-5*

**DOI:** 10.55730/1300-0152.2753

**Published:** 2025-05-20

**Authors:** Sümeyye AYDOĞAN TÜRKOĞLU, Aysu BOZKURT, Fatma POYRAZLI, Derya OKUYAN

**Affiliations:** 1 Department of Molecular Biology and Genetics, Faculty of Science and Literature, Balıkesir University, Balıkesir, Turkiye; 2 Department of Molecular Biology and Genetics, Institute of Science, Balıkesir University, Balıkesir, Turkiye; 3 Department of Veterinary Medicine, Susurluk Agriculture and Forestry Vocational School, Bandırma Onyedi Eylül University, Balıkesir, Turkiye

**Keywords:** Carbonic anhydrase VII, hypoxia, colon cancer, prostate cancer, *Caenorhabditis elegans*

## Abstract

**Background/aim:**

A number of carbonic anhydrase (CA) family proteins have been implicated in cancer. They contribute to the hypoxic microenvironment. CAVII is often downregulated in colorectal carcinoma and it has been associated with increased tumor size, node metastasis, and adverse clinical outcomes. In this study, we aimed to investigate the effect of hypoxia on CAVII protein in human colon cancer and prostate cancer cells. In addition, the regulation of *CAH* genes in *Caenorhabditis elegans* was examined. These are homologous to *CAVII* in humans.

**Materials and methods:**

CAVII expression was analyzed in different cell lines such as human colon cancer (SW480 and HT-29), human prostate cancer (PC3 and LNCaP), human hepatocellular carcinoma (Hep3B) and Human umbilical vein endothelial cells (HUVEC). HT-29 and LNCaP cell lines were subjected to a chemical hypoxia model with CoCl_2_. Real-time PCR was used for *CAVII* mRNA analysis. Western blot and immunofluorescence (IF) staining were used to detect the CAVII protein. The response of *CAH* genes was also studied at the mRNA level in a chemical hypoxia model with sodium sulfite in *C*. *elegans*. *CAVII* and *CAVII*-like genes *CAH-3*, *CAH-4*, and *CAH-5* were analyzed bioinformatically.

**Results:**

We found that CAVII expression decreased under hypoxic conditions in HT-29, but conversely, increased in LNCaP cells at the mRNA and protein level. In the hypoxia model in *C*. *elegans*, *CAH* genes were downregulated. According to bioinformatics analyses, human *CAVII* was most similar to *CAH-3* (98%).

**Conclusion:**

The results emphasize the necessity of addressing hypoxic regulation in different cell and organism groups in cancer and healthy conditions for CA family members that change under physiological and pathophysiological conditions. Postgenomic studies are important to better understand the evolution of these ancient enzymes.

## 1. Introduction

Carbonic anhydrases (CAs) catalyze reversible reactions of carbon dioxide hydration and dehydration, and have important roles in metabolic processes by regulating acid-base and ion levels. CAs are classified into 4 groups: cytosolic (CAI, CAII, CAIII, CAVII, CAVIII, CAX, CAXI, and CAXIII), mitochondrial (CAVA, CAVB), secreted (CAVI), and membrane-bound (CAIV, CAIX, CAXII, CAXIV, and CAXV) (Hewett-Emmett and Tashian, 1996). These isoforms differ in domain organization, enzyme activity, and degree of inhibition by CA inhibitors (Bottcher et al., 1994). Cytosolic CAs are expressed in all human tissues, have conserved enzymatic activity, and have a variety of functions. They maintain blood pH by producing HCO_3_^−^ (Kummola et al., 2005).

CAVII was first isolated and characterized by Montgomery et al. (1991) and is located on the long arm of 16q22. Other cytosolic CAs are located at the 8q21 locus in humans. The human *CAVII* gene is approximately 10 kb in length and contains 6 introns and 7 exons (Hewett-Emmett and Tashian, 1996). CAVII mRNA has been found in the human salivary gland (Montgomery et al., 1991), mouse hippocampus and cerebellum (Lakkis et al., 1996, 1997), and rat lung (Ling et al., 1994). The inhibition profile of CAVII is quite different to other cytosolic isoenzymes and does not show a stable affinity in the presence of inhibitors (e.g., acetazolamide, methazolamide, ethoxzolamide, and sulfonamide), and there is a need to design more selective inhibitors (Vullo et al., 2005). CAVII is the isoform with the highest esterase activity in the CA family (Supuran, 2008).

*CAVII* expression may have prognostic value in colorectal carcinoma (CRC). CAVII is often downregulated in CRC tissues at the mRNA and protein levels. Low *CAVII* expression in CRC has been associated with increased tumor size, node metastasis, and adverse clinical outcome. This difference in expression status suggests that *CAVII* can be used as an independent prognostic indicator in early stage CRC patients (Bootorabi et al., 2011, Yang et al., 2015). *CAVII* expression increases in various cancer types e.g., ovarian, gastric, astrocytomas (tumor formation that begins in astrocytes, cells that provide support to nerve cells), oligodendrogliomas (tumors formed by oligodendrocytes), and mixed oligoastrocytomas (tumors formed by oligodendroglioma and astrocytoma cells), while *CAVII* expression decreases in CRC (Yang et al., 2015). Inconsistent results regarding the prognostic value of CAVII in various malignancies suggests that its prognostic value may be tissue-dependent and varies according to the malignancy. The reason why CAVII is downregulated in CRC is not known and must be investigated. However, the results in CRC suggest that the determination of intratumoral CAVII expression status may help identify patients who have aggressive forms of CRC and may also guide individualized treatment options.

CAVII expression was found to be low in tumor tissues compared to other CAs, but CAVII expression was higher in thyroid carcinoma, colorectal adenocarcinoma, and low-grade brain glioma. Especially in colorectal adenocarcinoma, high CAVII expression was associated with poor prognosis and was suggested as a marker. In a bioinformatics study using the cBioPortal with *CAVII*, the largest change in CAVII expression was detected in breast cancer tissues, followed by deletions in malignant peripheral nerve tumors and mutations in desmoplastic melanoma (Cerami et al., 2012).

In an immunohistochemistry (IHC) study of CAVII, moderate cytoplasmic and low nuclear expression was detected, but high band profiles were obtained in several ovarian and gastric cancer cases studied (Uhlen et al., 2017). Bootorabi et al. (2011) examined CAVII expression in 3 brain tumor categories: astrocytomas, oligodendrogliomas, and mixed oligoastrocytomas. The findings showed that CAVII expression was higher in tumors that spread to the body via the lymph or blood. Their results indicate that high CAVII expression might be a marker of poor prognosis in patients who have astrocytoma-derived brain tumors. They have shown that CAVII might be another CA isoenzyme that has a significant correlation with patient survival. The presence of several CA isoenzymes in astrocytomas may provide a rapid turnover of acidic metabolic products in highly malignant tumor tissues. Cytosolic isoenzymes e.g., CAII and CAVII may contribute to more efficient neutralization of the cell interior, while membrane-associated enzymes CAIX and CAXII participate in the export or removal of protons together with membrane ion transport proteins. These mechanisms may provide new opportunities in cancer treatment, where the tumor cell microenvironment can be targeted by CA inhibition. Research has focused on several compounds as highly efficient CA inhibitors (Pastoreková et al., 1992). Some drugs can inhibit neoangiogenesis while reducing tumor growth, and some compounds may also reduce the invasive capacity of tumor cells (Supuran and Scozzafava, 2001; Supuran et al., 2003).

Hypoxia is a condition induced by the lack of oxygen in tissues and cells. Cells that are deprived of oxygen try to receive oxygen from various cells and tissues through vascularization. This is commonly seen in cancer cells. The hypoxic microenvironment in which tumors are located provides resistance to various chemotherapeutic drugs. Because of the rapidly proliferating nature of cancer cells, the tumors that form rapidly consume nutrients and oxygen, making the environment hypoxic. Angiogenic factors secreted from tumors in the hypoxic environment trigger vascularization and tumor diameter growth. In short, the surroundings of solid tumors are hypoxic areas. Structures deprived of oxygen move away from their environments through angiogenesis (Aydoğan Türkoğlu and Köçkar, 2012; Aydoğan Türkoğlu et al., 2021). Hypoxia-inducible factors (HIFs) are transcription factors that play a role in the carcinogenesis process. HIF expression increases in tumor tissues (Goldberg et al., 1988). In hypoxic conditions, the adenosine monophosphate activating protein kinase (AMPK) pathway and the HIF pathway are activated. HIF is responsible for sensing the oxygen level in the environment where the cells are located. The AMPK pathway is activated when the amount of ATP in the cell decreases and activates catabolic processes while inhibiting anabolic processes. Cancer cells have developed various ways to activate HIF by inactivating tumor suppressor genes, e.g., *PTEN* and *VHL*, activating various oncogenes, e.g., *c-MYC*, and increasing the activation of various growth factor pathways, e.g., insulin-like growth factor 1 (IGF-1), IGF-2, and platelet-derived growth factors (PDGF) (Maxwell et al., 2001). Under hypoxic conditions in cells, pathways like vascular endothelial growth factor (VEGF), fibroblast growth factor (FGF), erythropoietin, endothelin-1, and PDGF are also activated along with HIF (Aydoğan Türkoğlu et al., 2021).

In a study conducted to elucidate hypoxia-induced CA-mediated dorsal horn neuron activation and induction of neuropathic pain, it was shown that hypoxic conditions resulted in increased expression of *CAVII* in neurons isolated from the lumbar region of the spinal cord (Lobo et al., 2022). Many studies have explored *CAIX*, *CAXII*, *CAI*, and *CAII* genes. However, studies on the *CAVII* gene, especially on its expression in hypoxic conditions, are limited. Yildirim et al. (2017) reported that TGF-β cytokine increased *CAIX* expression in Hep-3B cells under hypoxic conditions. Hypoxia-induced proteins are important targets for anticancer therapy. Understanding the mechanisms underlying the TGF-β-mediated upregulation of *CAIX* in hepatoma cells allows new treatment strategies to be developed for hepatocellular carcinoma (Yildirim et al., 2017). Okuyan et al., (2020) found that when hypoxic conditions were mimicked in a prostate cancer cell line, *CAIII* expression increased at the mRNA and protein level.

Functional studies suggest that CAs are not only involved in regulating pH homeostasis but also contribute to many previously unidentified functions. For the past 3 decades, mice, zebrafish, fruit flies, and *Caenorhabditis elegans* have been primary model organisms for studying a variety of biological phenomena. Moreover, since *C*. *elegans* was the first multicellular organism to have its genome sequenced, it is a particularly useful model for identifying new key genes in fundamental biological processes. In our study, in addition to examining the regulation of the *CAVII* gene in human cancer cell line, we created a hypoxic *C*. *elegans* model and investigated the regulation of *C*. *elegans* CA-like genes (*CAH-3*, *CAH-4*, and *CAH-5*) in this model. Seven α-CAs are encoded by the *C*. *elegans* genome: *CAH-1*, *CAH-2*, *CAH-3*, *CAH-4a*, *CAH-4b*, *CAH-5*, and *CAH-6*. In our bioinformatics studies, *CAH-3*, *CAH-4*, and *CAH-5* showed the highest similarity with human *CAVII*, so these genes were included in the study. Furthermore, these three *C*. *elegans* CAs are cytoplasmic and carry Zn in their active sites, much like human CAs (Aspatwar et al., 2022). Furthermore, we monitored the expression of *HF1A* and *Ce-HIF* in human cells and *C. elegans*, respectively, to confirm the presence of hypoxia.

In this study, we aimed to investigate the effect of hypoxia on the CAVII protein. The pH of the tumor microenvironment is one of the most common effects of hypoxia. The extracellular pH of tumor cells is more acidic than that of normal cells due to an increase in the production of lactic and carbonic acids. Due to the highly variable oxygen levels within tumors, hypoxia can range from mild to severe during certain pathophysiological events, such as cancer. A poor prognosis is caused by tumor hypoxia that is linked to increased radio resistance and the possibility of metastasis (Höckel et al., 2001; Harris, 2002; Bussink et al., 2003). This is a pioneering study to elucidate CAVII regulation in hypoxia.

## 2. Materials and methods

### 2.1 Cell culture and hypoxia treatment

Human colon cancer (SW480 and HT-29), human prostate cancer (PC3 and LNCaP), human hepatocellular carcinoma (Hep3B) and Human umbilical vein endothelial cells (HUVEC) were grown in DMEM (Dulbecco’s modified Eagle medium) (Sigma-Aldrich, St. Louis, MO, USA), enhanced with 10% fetal calf serum (Invitrogen, Waltham, MA, USA) that had been heat-inactivated (56 °C for 1 h) and 2 mM L-glutamine (HyClone, Logan, UT, USA). The cells were kept in a 5% (v/v) CO_2_ environment at 37 °C. Trypan blue exclusion was used to assess the viability of the cells before the experiment. In 5 mL of media, the cells were passaged into a 25 cm^2^ area (2 million cells per flask). To help the cells adhere to the flask surface, they were incubated overnight. HT-29 and LNCaP cells were exposed to a final concentration of 150 μM of CoCl_2_ for 24, 48, and 72 h, to induce a hypoxic effect in the cells (Aydogan Turkoglu and Kockar, 2016).

### 2.2. RNA isolation and verification of cDNAs

An RNA isolation kit (Thermo Fisher, Scientific, Waltham, MA, USA) was used to isolate total RNA from cell pellets. RNA as kept in the freezer for extended periods at −80 °C. Reverse transcriptase (RT) PCR was performed in two stages. Reverse transcriptase (RT) was used in the first stage to synthesize cDNA. Using cDNA from the first stage and gene-specific primers, amplification of the gene area and polymerase chain reactions (PCRs) were performed in the second stage. Table shows the primer sequences for *CAVII, β-2 microglobulin (β2M)*, and hypoxic-inducing factor 1-α *(HIF1α)*.

PCR results were examined using a gel-doc system after being run on a 1% (w/v) agarose gel. Using the Image J image processing program, densitometric analyses were carried out, and *CAVII* expression was normalized in relation to *β2M* expression (Okuyan et al., 2020).

### 2.3. Quantitative real-time PCR

The mRNA expression of CAVII was measured using real-time PCR in normoxic and hypoxic environments. For real-time PCR, 1 μL cDNA, 5 μL SYBR Green PCR Master Mix, 0.5 μL forward and reverse primer (50 ng/μL), and 3 μL dH2O were added to 96-well plates to fill them to a final volume of 10 μL. For every cDNA sample, three replications of *CAVII and HIF1α* were carried out. As an internal control, three replications were carried out using β2M primers. The Livak technique was used to analyze the real-time PCR results. Minitab 14 was used for statistical analysis (Okuyan et al., 2020).

### 2.4. Western blot analysis

In the radio-immunoprecipitation assay (RIPA) buffer, cells were lysed. A Bradford curve was created to calculate the quantities of protein. A 10% polyacrylamide gel was used to run 30 μg of protein extract. The gel was then transferred to polyvinylidene difluoride (PVDF) membranes (Millipore Sigma, Burlington, MA, USA). To avoid nonspecific binding, membranes were then blocked in 1X tris-buffered saline (TBS) that had 5% (w/v) nonfat milk powder and 0.1% (v/v) Tween-20. Membranes were initially incubated for 12–18 h at 4 °C with CAVII primary antibody (Invitrogen, PA5-103703). Following three cycles of washing, the membranes were incubated for 1 h with a secondary antibody (HRP-conjugated antimouse). Membranes were transferred on film after being treated with Pierce’s chemiluminescence substrate. Markers were used to analyze the bands. The bands were examined using the Image J program. The amount of β-actin protein was used to normalization and the amount of CAVII protein was recorded (Okuyan et al., 2020).

### 2.5. Immunofluorescence staining

For immunofluorescence (IF) staining HT-29 and LNCaP cells were cultivated in 24-well plates containing DMEM and 125 × 10^3^ cells per well. Cells were exposed to a final concentration of 150 μM of CoCl_2_ for 24 h. Untreated cells were used as a reference. The cells were preserved for 15 min at room temperature in phosphate buffer saline (PBS) containing 4% paraformaldehyde. After applying a blocking solution containing 1% BSA in PBS for 30 min, the cells were treated with a 1 in 50 dilution of anti-CAVII polyclonal antibody and kept at 4 °C overnight. The cells were then treated at 25 °C for 1 h with the Alexa Fluor 488 secondary antibody. DAPI staining was applied to the cells following incubation with the second antibody and the cells were examined using an Olympus DP72 fluorescent microscope (Tokyo, Japan) (Poyrazlı et al., 2024).

### 2.6. *C. elegans* culture and hypoxic conditions

A wild type *C*. *elegans* strain and *Escherichia coli* OP50 strain were obtained from the Caenorhabditis Genetics Center (CGC) University of Minnesota. Fresh nematode growth medium (NGM) agar plates containing *E*. *coli* OP50 were plated with *C*. *elegans*, and the plates were incubated for 3 days at 20 °C. Sodium sulfite solutions were made in M9 buffer at three different concentrations (0.5, 1.0, and 2.0 g/L) to simulate hypoxia. On the third day, *C*. *elegans* was cultured at 26 °C for varying durations after being rinsed three times with M9 buffer and moved into 1.5 mL tubes with 1 mL of fresh incubation solution. The organisms were moved to NGM plates after incubation so they could recover for 24 h at 20 °C (Sutphin and Kaeberlein, 2009).

### 2.7. Total RNA isolation from *C. elegans* and real-time PCR

After being removed from the medium and cleaned with M9 buffer, the organisms were put in a Falcon tube and left on ice for 1 h. After aspirating the M9 on the organisms, 5 mL of M9 buffer was added to the tubes that were then placed on ice for 15 min. The organisms were transferred into 1.5 mL tubes after the M9 buffer was removed by pipetting. A pellet was created by centrifuging the tubes. After removing the supernatant, the pellet was mixed with 1 mL of Trizol. The tubes were rapidly frozen by submerging them in liquid nitrogen, and they were thawed at 37 °C five times. The samples were then centrifuged for 30 s at 3200 rpm and 200 μL of chloroform was added. They were left to incubate for 15 min at room temperature. Centrifugation was carried out for 15 min at 12,000 rpm. After transferring the aqueous phase to a fresh tube, 500 μL of isopropyl alcohol was added. After 10 min of room temperature incubation, the samples were centrifuged for 10 min at 12,000 rpm. After careful removal, the supernatant was transferred to a sterile tube. To the supernatant, 1 mL of 75% ethanol was added and the samples were centrifuged for 5 min at 7500 rpm. The pellet was then allowed to dry after the supernatant was discarded. The pellets were dissolved in 30–50μL of ddH_2_O. The quantity and quality of RNA were determined by spectroscopy (Greer Lab. 2012)^1^.

A commercial kit was used for cDNA synthesis. PCR Reactions were performed in a final volume of 10 μL, with 5 μL RealQ Plus 2X Master Mix Green (Ampliqon, Odense, Denmark) and 0.5 μL of each primer pair (10 ng/μL). The *Ce-HIF* primer was used to validate the presence of hypoxia. *CAH-3* (NC_003284.9), *CAH-4* (NC_003284.9), and *CAH-5* (NC_003284.9) sequences were used in the primer design of *C*. *elegans* CA-like genes. Table shows the primer sequences for the, *Ce-HIF*, *CAH-3*, *CAH-4*, and *CAH-5* genes. In addition, the *CDC-42* gene was used for normalization. The 2^−ΔΔCt^ values were used to determine fold changes in the mRNA expression of *CAH-3*, *CAH-4*, *CAH-5*, and *Ce-HIF* were determined (Jiang et al., 2011).

### 2.8. Bioinformatic analysis

The similarity of *Homo sapiens CAVII* and *C*. *elegans CAH* genes were analyzed using BioEdit and NCBI blast^2^ programs. STRING database^3^ was used to draw the associated protein network diagram of human CAVII and *C*. *elegans CAH* genes. Further analysis was performed with the KEGG database to display the basic biological processes the proteins are involved in.

### 2.9. Statistical analysis

Determination of *HIF1α*, *CAVII*, *Ce-HIF*, *CAH-3*, *CAH-4*, *CAH-5* mRNA levels were performed in human cell lines and *C*. *elegans* in independent experiments at different times. *HIF1α* and *CAVII* mRNA levels were normalized by comparing with *β2M* and averaged from three replicate groups. *CAH* mRNA levels were normalized with *CDC42* and averaged from three replicate groups. Ct values obtained from real-time PCR were analyzed according to Livak method. The results were visualized using GraphPad Prism 8. The results were evaluated statistically with one-way ANOVA (p < 0.05 was considered significant). The experiment was carried out in three replications.

## 3. Results

### 3.1. Determination of CAVII expression in different human cell lines

**Figure 1:**
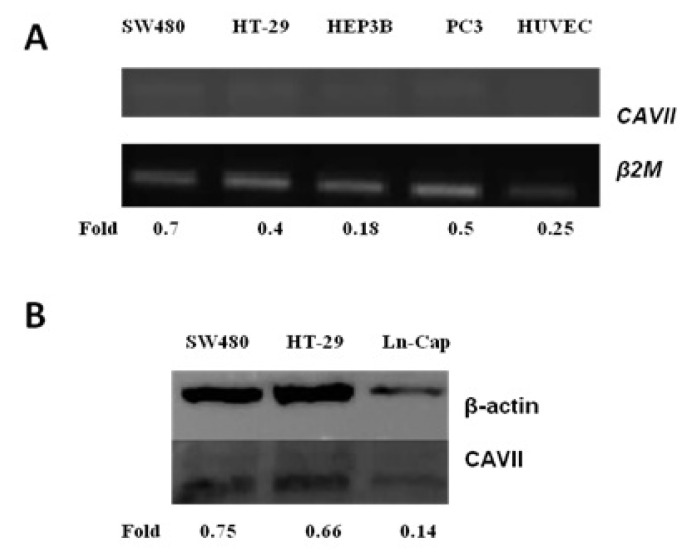
CAVII expression in different cell lines. (A) Densitometric analysis of *CAVII* mRNA expression in SW480, HT-29, Hep3B, PC3, and HUVEC cells. (B) Densitometric analysis of CAVII protein expression in SW480, HT-29 and LNCaP cells.

Human *CAVII* gene expression was examined in human colon cancer (SW480 and HT-29), human prostate cancer (PC3 and LNCaP), human hepatocellular carcinoma (Hep3B) and Human umbilical vein endothelial cells (HUVEC) with primers designed on the NM_005182.2 sequence (Table). The designed primers amplified a 287 bp region. As seen in Figure 1A, *CAVII* mRNA expression was detected in the prostate cancer cell lines (PC-3 and LNCaP), colon cancer cell lines (SW480 and HT-29), hepatocellular carcinoma cell line (Hep3B), and healthy cell line (HUVEC). Additionally, protein levels were analyzed in SW480, HT-29 and LNCaP cell lines by western blot analysis (Figure 1B).

### 3.2. Response of CAVII expression to hypoxia in a colon cancer cell line

**Figure 2:**
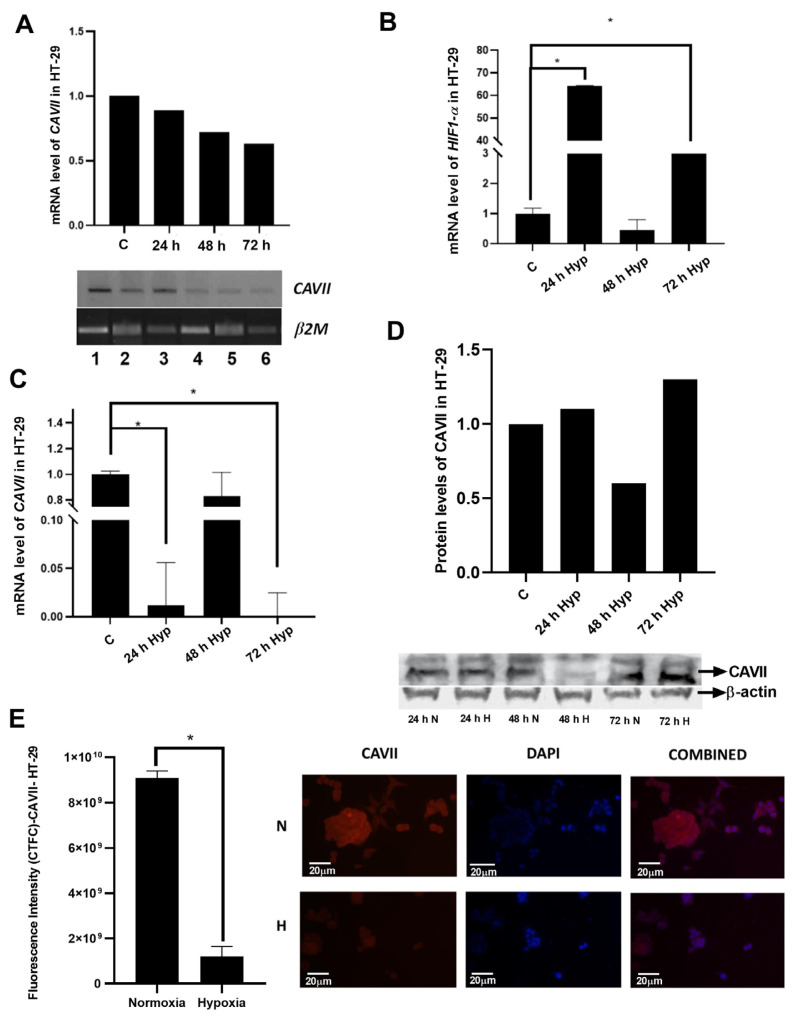
Changes in CAVII expression in HT-29 cell line under normal and hypoxic conditions. A) Densitometric analysis of *CAVII* level by real-time PCR at mRNA level. B) Analysis of *HIF1α* mRNA level by real-time PCR for detection of hypoxic conditions. C) Analysis of *CAVII* mRNA level by real-time PCR under normal and hypoxic conditions. D) Determination of CAVII protein level by western blot under normal and hypoxic conditions. E) Determination of CAVII protein level by IF staining under normal and hypoxic conditions. *indicates statistical significance (p < 0.05).

HT-29 colon cancer cells were incubated in normoxic and hypoxic conditions for 24, 48, and 72 h. Chemical hypoxia was induced in this cell line with CoCl_2_. In the chemical hypoxia model, the *CAVII* mRNA level was evaluated densitometrically (Figure 2A). Hypoxic conditions were confirmed by monitoring the expression of *HIF1α* (Figure 2B), the main regulatory protein of hypoxic conditions. The mRNA level of *CAVII* was also evaluated by real-time PCR (Figure 2C). Changes in CAVII protein expression were evaluated by western blot (Figure 2D) and IF staining (Figure 2E).

The level of *HIF1α*, the main regulator of hypoxia, significantly increased in the 24 and 72-h hypoxic groups in the HT-29 cell line compared to the control (p < 0.05) (Figure 2B). In densitometric analysis, *CAVII* expression decreased at all time points compared to the control group (Figure 2A). Real-time PCR was performed to confirm hypoxia and to determine *CAVII* mRNA expression more sensitively, showing *CAVII* mRNA expression significantly decreased compared to normal conditions at all 24- and 72-h time points (Figure 2C). The CAVII protein level was analyzed by western blot and IF staining. CAVII bands were normalized with β-actin in western blot analyses. The results showed a very strong decrease in CAVII at 48 h (Figure 2D), and the IF staining results supported this finding (Figure 2E).

### 3.3. Response of CAVII expression to hypoxia in a prostate cancer cell line

**Figure 3:**
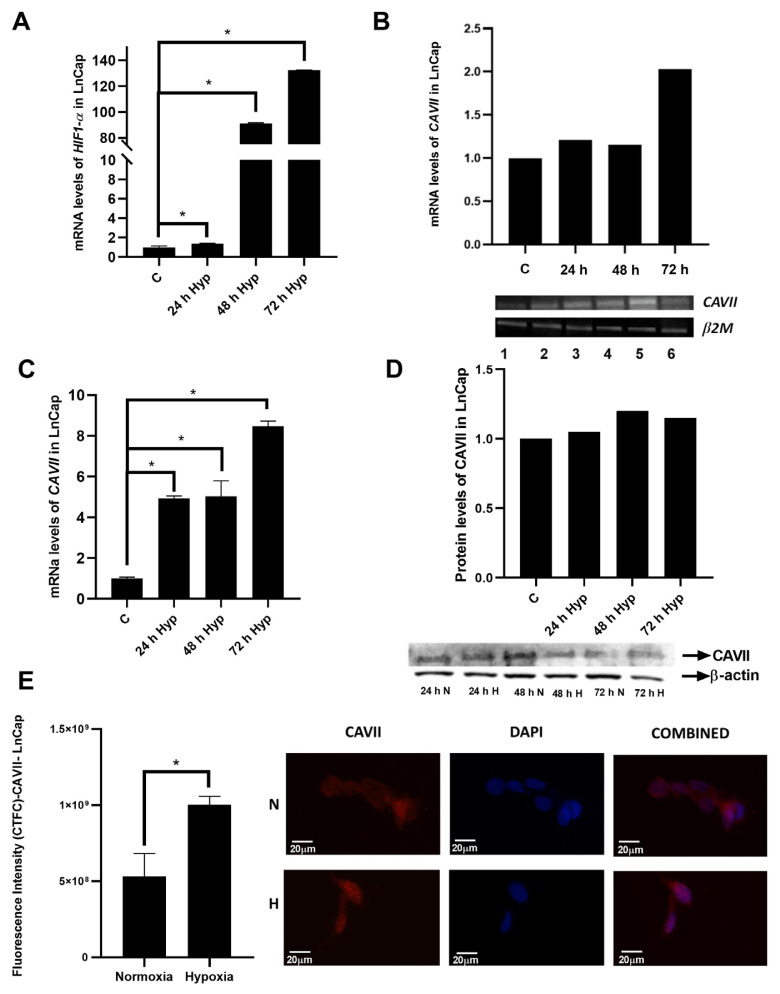
Changes in CAVII expression in the LNCaP cell line under normal and hypoxic conditions. A) Densitometric analysis of *CAVII* level by real-time PCR at mRNA level. B) Analysis of *HIF1α* mRNA level by real-time PCR for detection of hypoxic conditions. C) Analysis of *CAVII* mRNA level by real-time PCR under normal and hypoxic conditions. D) Determination of CAVII protein level by western blot under normal and hypoxic conditions. E) Determination of CAVII protein level by IF staining under normal and hypoxic conditions. *indicates statistical significance (p < 0.05).

To analyze whether the CAVII hypoxic response is specific to cancer, LNCaP cells were tested. First, hypoxic conditions were created in this cell line and confirmed by monitoring *HIF1α* expression. As seen in Figure 3A, *HIF1α* mRNA levels significantly increased in 24-, 48-, and 72-h periods compared to normal conditions. *CAVII* mRNA expression in LNCaP cells was evaluated densitometrically (Figure 3B) and by real-time PCR according to the Livak method (Figure 3C). *CAVII* mRNA expression significantly increased in hypoxic conditions at all time points compared to the control (Figure 3C). There was a lower level of increase in the CAVII protein expression levels in western blot (Figure 3D) and IF staining (Figure 3E).

### 3.4. Bioinformatic analysis of human *CAVII* and *C. elegans CAH* genes

**Figure 4:**
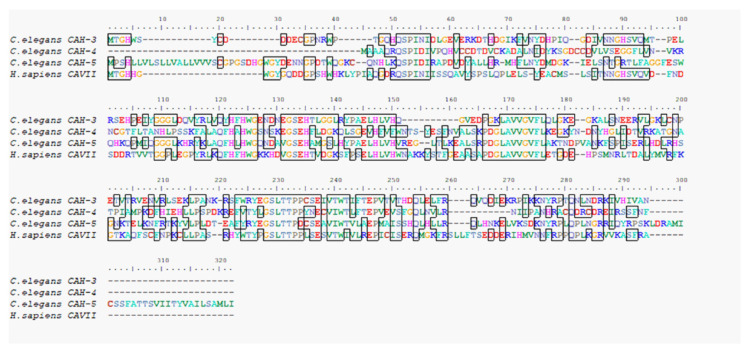
Amino acid sequence comparison of human *CAVII* and *C*. *elegans CAH-3*, *CAH-4*, and *CAH-5* genes. Conserved regions in the compared sequences are framed.

Since *CAH-3*, *CAH-4*, and *CAH-5* showed the highest similarity with human *CAVII* in our bioinformatic studies, these *C*. *elegans* genes were selected in our study. Furthermore, the proteins are cytoplasmic they carry Zn^+2^ in their active sites like human CAs. Hall et al. (2008) found sequence similarity between CAVII and CAH-3, CAH-4, CAH-5, and CAH-6 of 97.2%, 77.9%, 81%, and 76.2%, respectively. To find similarities between human CA genes and CAH-3, CAH-4, and CAH-5 genes in *C*. *elegans*, the amino acid sequences of all human CAs (CAI–CAXII), and CAH-3, CAH-4, CAH-5 genes were aligned in BioEdit. Figure 4 shows comparisons of human CAVII amino acid sequence with selected *C*. *elegans* CAH amino acid sequences.

In our NCBI analyses on human *CAVII*, three isoforms of this gene were recorded. Primers specific to variant 1 were designed and used in our study. Variant 1 (NM_005182.3) encodes the longest isoform (258 aa) although it represents a shorter transcript (1518 bp mRNA). Variant NM_001014435.2 (1710 bp mRNA) has a different 5′ UTR and uses a different start codon compared to variant 1. It encodes isoform 2, which has a shorter N-terminus compared to isoform 1 (208 aa). Another variant registered with NCBI, NM_001365337.2 (1514 bp mRNA), appears to have a different 5′ UTR and uses a different start codon compared to variant 1 (208 aa). It encodes isoform 2, which has a shorter N-terminus compared to isoform 1. Variants 2 and 3 registered in NCBI actually encode the same 208 amino acid protein.

The ORF of the human *CAVII* gene is 1518 bp long and encodes a polypeptide of 264 amino acids. The predicted polypeptide was a 29.6584 kDa protein with a pI of 6.92. The polypeptide had an aliphatic index of 67.92 and the grand average of hydropathicity (GRAVY) of −0.487, indicating that the human CAVII protein is hydrophilic. While the total number of negatively charged residues (Asp + Glu) is 27, the total number of positively charged residues (Arg + Lys) is 26.^4^

Since the highest similarity with human *CAVII* was with the *C*. *elegans CAH-3* gene, further analyses were carried out with the NM_001383694.2 sequence (1080 bp mRNA). The predicted polypeptide was a 29.6584kDa protein with a pI of 5.70. The polypeptide had an aliphatic index of 77.52 and the grand average of hydropathicity (GRAVY) of −0.731, indicating that the CAH-3 protein is hydrophilic. While the total number of negatively charged residues (Asp + Glu) is 36, the total number of positively charged residues (Arg + Lys) is 26 (Gasteiger et al., 2005).

When we compare histidine residues in particular, human CAVII and CAH-3 carry a conserved histidine at position 90. When the other histidine residues were examined, positions 122, 124, and 130 are conserved in human and *C*. *elegans CAH* genes. This pattern of conserved histidine residues across species is noteworthy because histidine residues are very important for the activity of CAs.

### 3.5. Expression analysis of *CAVII* homologous *CAH* genes in the *C. elegans* model

**Figure 5:**
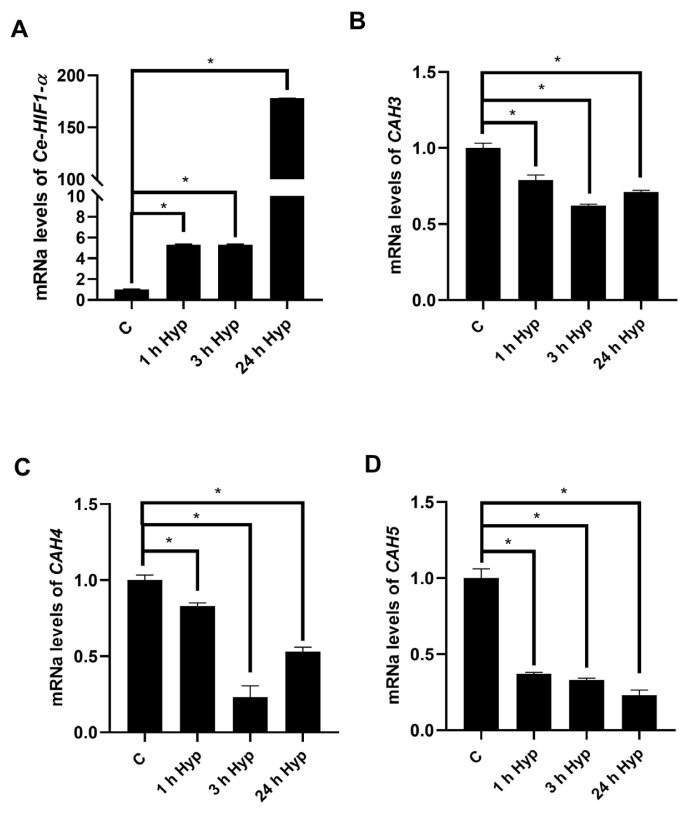
Validation of *C*. *elegans* hypoxic model and analysis of *C*. *elegans CAVII*-like genes. A) Comparison of *Ce-HIF* expression in normal and hypoxic conditions, B) *CAH-3* mRNA expression, C) *CAH-4* mRNA expression, D) *CAH-5* mRNA expression.

Unlike human studies, sodium sulfite was used as the chemical hypoxia agent in *C*. *elegans*. The expression level of the hypoxia regulator gene *Ce-HIF* was analyzed to confirm hypoxia in the *C*. *elegans* model. The exposure conditions in the *C*. *elegans* model were different to the human cell line experiments because *C*. *elegans* is a very different organism. Hypoxia was created in *C*. *elegans* at 1, 3, and 24 h, and *C*. *elegans* without application was used as a control group. The validity of this novel hypoxic model was confirmed by the significant increase in *Ce-HIF* expression in all conditions (Figure 5A).

After confirming the hypoxic conditions, the expression of *C*. *elegans CAH-3*, *CAH-4*, and *CAH-5* genes, which are human *CAVII*-like genes, was analyzed by real-time PCR method under hypoxic conditions than normal conditions. Expression of *CAH-3*, *CAH-4*, and *CAH-5* genes significantly decreased at all time points in hypoxic conditions compared to the control group (Figure 5B–5D).

### 3.6. Pathways in which the human *CAVII* and *C. elegans CAH-3, CAH-4*, and *CAH-5* genes are involved and genes they interact with

**Figure 6:**
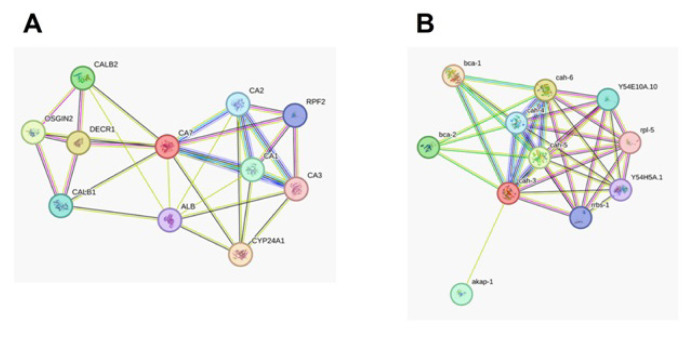
A. Pathway analysis of the human CAVII (A) CYP24A1: 1,25-dihydroxyvitamin D(3) 24-hydroxylase, mitochondrial; DECR1: 2,4-dienoyl-CoA reductase; OSGIN2: Oxidative stress-induced growth inhibitor 2; CALB2: Calretinin; Calretinin is a calcium-binding protein; CA1: Carbonic anhydrase 1; CA2: Carbonic anhydrase 2; CA3: Carbonic anhydrase 3; CALB1: Calbindin; Buffers cytosolic calcium; RPF2: Ribosome production factor 2 homolog; ALB: Serum albumin. B. Pathway analysis of the C. elegans CAH (B) genes. bca-1: Beta carbonic anhydrase 1; bca-2: Beta carbonic anhydrase 2; cah-4: Alpha-carbonic anhydrase domain-containing protein; cah-5: Putative carbonic anhydrase 5; cah-6: Alpha-carbonic anhydrase domain-containing protein; akap-1: KH domain-containing protein akap-1; Y54E10A.10: Ribosome production factor 2 homolog; rrbs-1: Ribosome biogenesis regulatory protein homolog; Y54H5A.1: WD_REPEATS_REGION domain-containing protein; rpl-5: 60S ribosomal protein L5.

The genes selected in both models were related to the G protein-coupled receptor (GCPR) signaling pathway, cellular response to dopamine, chemical synaptic transmission, cellular response to chemical stimulation, regulation of multicellular organism processes, serotonin binding, G protein-coupled serotonin receptor activity, neurotransmitter receptor activity, and GPCR ligand binding signaling pathways (Figures 6A and 6B).

Pathway analyses showed that *C*. *elegans* CAH-3 may function together with other family members, especially CAH-4, CAH-5, and CAH-6. CAH-3 has been shown to be associated with ribosome-related pathways. CAH-3 may also be associated with beta group CAs. STRING analysis on human CAVII signaling pathways showed that CAVII may be associated with CAI, CAII, and CAIII in CA family members and may be associated with oxidative stress-related pathways (Figure 6A). The ribosome relationship seen in *C*. *elegans* analyses was also associated with the RPF2 signaling pathway in human CAVII signaling pathway analyses (Figure 6B).

## 4. Discussion

Of all CA isoforms, CAVII expression is lower in healthy tissues and tumors. According to human genome atlas data, *CAVII* expression is found in the brain, digestive system organs, kidney, lung, colon, and testes. In the literature, *CAVII* gene expression has been examined in tissue samples taken from various brain tumors, colon cancer, stomach cancer, and ovarian cancer individuals. In particular, Yang et al. (2015) found that *CAVII* expression decreased in colon cancer tissues and this led to cancer progression (Bootorabi et al., 2011; Yang et al., 2015, Uhlen et al., 2017). Hypoxia is the lack of oxygen to the tissues that occurs in many diseases, especially cancer. This leads to a decrease in oxygen in normal tissues. The hypoxic microenvironment that occurs in normal tissues causes proliferation. HIFs bind to target sequences in the promoter and affect the expression of many genes (Goldberg et al., 1988). Although *HIF1α* is overexpressed in most types of cancer in humans, the role of this gene in cancer formation is not fully understood. In our study, the response of CAVII, an important enzyme for cellular physiology, to hypoxia at the mRNA and protein levels was investigated in two human cell line models.

CAVII expression is downregulated at mRNA and protein levels in CRC. Low CAVII expression in CRC has been associated with increased tumor size, node metastasis, and adverse clinical outcome. This difference in expression status suggests that CAVII can be used as an independent prognostic indicator in early-stage CRC patients (Bootorabi et al., 2011). CAVII expression in tumors is lower than most CA isoforms and is highest in thyroid carcinoma, colorectal adenocarcinoma, and low-grade brain gliomas. In colorectal adenocarcinoma, CAVII upregulation may serve as a marker for poor prognosis. In a bioinformatics study with CAVII via the cBioPortal, the highest change in CAVII expression was detected in breast cancer tissues, followed by deletions in malignant peripheral nerve tumors and mutations in desmoplastic melanoma (Cerami et al., 2012). In an IHC study of CAVII, moderate cytoplasmic and less nuclear expression was detected, but high band profiles were obtained in several ovarian and gastric cancer cases studied (Uhlen et al., 2017). Studies on CRC at the mRNA and protein levels have shown that CAVII is often downregulated in CRC and this is closely associated with aggressive clinical characteristics and poor postoperative prognosis in CRC patients. Yang et al.(2015) found that *CAVII* expression increases in other types of cancer but decreases in CRC. Inconsistent results regarding the prognostic value of CAVII in various malignancies show that its prognostic value might be tissue-dependent varying according to malignancy. The mechanism underlying the prognostic significance of CAVII in CRC is not known and requires investigation.

The prognostic value of various CA members and their importance in cancer regulation have been determined. IHC analyses have shown that CAIX and CAXII are widely expressed in many normal tissues and epithelial tumors and that this expression level increases under hypoxic conditions (Ivanov et al., 2001). CAIX and CAXII expression increases in cells in the hypoxic medium in brain tumors of various malignancies. The increased expression level indicates that hypoxia is an induction factor (Proescholdt et al., 2005). Okuyan et al. (2020) found that hypoxic condition induced CAIII mRNA and protein expression in a prostate cancer cell line (Okuyan et al., 2020). Sarnella et al. (2022) found increased *CAIX* and *CAXII* gene expression in head and neck squamous carcinoma cells grown under hypoxic conditions. However, the addition of SLC-0111, a CAIX and CAXII inhibitor, reduced tumor growth and spread (Sarnella et al., 2022). Studies on the *CAVII* gene specifically are limited. Although there are studies on *CAVII* in astrocytomas, oligodendrogliomas, oligoastrocytomas, ovarian cancer, stomach cancer, and colon cancer tumors, no study has been found to elucidate CAVII regulation in other cancer types and cancer cell lines.

In our study, we determined the hypoxic response of CAVII in two selected cancer cell lines (HT-29/LNCaP). We found that CAVII expression decreased under hypoxic conditions in HT-29 cells. These data supports the downregulation of CAVII in colon cancers in the literature. We conclude that CAVII expression decreases as a result of increased hypoxia in colon cancer. Hypoxia caused an increase in *CAVII* mRNA and protein levels in the prostate cancer cell line LNCaP. This means that the regulation of this gene can vary between different cancers. In our study, a different cell model, the *C*. *elegans* model, was used. Hypoxia was created with sodium sulfite in this organism and the mRNA levels of *CAVII*-like genes, *CAH-3*, *CAH-4*, and *CAH-5*, were analyzed with real-time PCR. A decrease in the expression of these genes was detected in hypoxic conditions during the time points studied.

The *C*. *elegans* results confirmed that CAs, which are genes regulated by hypoxia, are also regulated in this organism. Hypoxia decreased the expression of *CAVII*-like genes in this organism. All results emphasize the necessity of addressing hypoxic regulation in different cell and organism groups in cancer and healthy conditions for CA family members that change under physiological and pathophysiological conditions and have prognostic value.

In the study of CAs, which are physiologically very important enzymes, knock-in and knockout models have been created with mice, lower vertebrates, and invertebrate organisms. Unique animal models have been created and used to study the physiological role of each isoform. Postgenomic studies have produced a lot of genomic data to better understand the evolution of these ancient enzymes and to identify genetic variations that have physiological and clinical importance. In the last 20 years, studies on the relationship between CAs and diseases, and the development of specific CA inhibitors have been carried out intensively. CA inhibitors, for example, have anticancer and antimicrobial properties. In addition, the study of CA genes whose regulation is disrupted in many types of cancer has provided data for developing diagnostic marker genes in cancer studies.

This study shows that hypoxia and its cellular response are effective mechanisms in the regulation of different proteins in the evolutionary process, and that it is meaningful to study *CA-like* genes in *C*. *elegans* to show the effects of hypoxia at the cellular level as an alternative model. Determining the pathways and proteins through which hypoxia affects at the cellular level facilitates the understanding of pathophysiological processes in cancer, and develop new approaches for its treatment. Furthermore, this study shows that research on CAs is a promising area of research for future studies.

## Figures and Tables

**Table:** Sequences of primers used for PCR.

Name	Sequence
*CAVII* Forward	5′-CTGCTTTAAGAGGCTGCTCCG-3′
*CAVII* Reverse	5′- CCCTGGGCAATGGGATACAG-3′
*β2M* Forward	5′-TTTCTGGCCTGGAGGCTATC-3′
*β2M* Reverse	5′- CATGTCTCCATCCCACTTAACT-3′
*HIF1-α* Forward	5′- CCACCTATGACCTGCTTGGT-3′
*HIF1-α* Reverse	5′- TGTCCTGTGACTTGTCC-3′
*CAH-3* Forward	5′- AATCCGGAGACAGTGACTCGTG-3′
*CAH-3* Reverse	5′- AACGACCTCTTGTTTGCTGGC-3′
*CAH-4* Forward	5′- ACAGCTCAACGTGCTCCGTAA-3′
*CAH-4* Reverse	GGAAGATCGGATTTCACGGTC-3′
*CAH-5* Forward	5′-CACGATCTGAGACACTCTGGCA-3′
*CAH-5* Reverse	5′- AGAACGCTTCCGTGTCTAGTGG-3′
*CDC-42* Forward	5′- AGCCATTCTGGCCGCTCTCG-3′
*CDC-42* Reverse	5′- GCAACCGCTTCTCGTTTGGC-3′
*Ce-HIF* Forward	5′- CAACGTGTTTATGGGCAAAT-3′
*Ce-HIF* Reverse	5′- CATTGCAAAACGTCATCGTA-3′

## Footnotes

1 Greer Lab. (2012). Eric Greer. https://greerlab.wustl.edu/items/c-elegans-rna-extraction/ [accessed 01 March 2025].

2 BLAST (2025). Protein blast [online]. Website https://blast.ncbi.nlm.nih.gov/Blast.cgi?PROGRAM=blastp&PAGE_TYPE=BlastSearch&LINK_LOC=blasthome [accessed 01 March 2025].

3 STRING (2025). Multiple Proteins Search [online]. Website https://string-db.org/cgi/network?taskId=b6mdZ3Zfb2rj&sessionId=bKDfcvfHQKm4 [accessed 01 March 2025].

4 Expasy (2025). Protein Analysis [online]. Website https://web.expasy.org/protparam/ [accessed 01 March 2025].
